# DrugSig: A resource for computational drug repositioning utilizing gene expression signatures

**DOI:** 10.1371/journal.pone.0177743

**Published:** 2017-05-31

**Authors:** Hongyu Wu, Jinjiang Huang, Yang Zhong, Qingshan Huang

**Affiliations:** 1 School of Life Sciences, Fudan University, Shanghai, China; 2 Shanghai High-Tech United Bio-Technological R&D Co., Ltd., Shanghai, China; Universita degli Studi di Torino, ITALY

## Abstract

Computational drug repositioning has been proved as an effective approach to develop new drug uses. However, currently existing strategies strongly rely on drug response gene signatures which scattered in separated or individual experimental data, and resulted in low efficient outputs. So, a fully drug response gene signatures database will be very helpful to these methods. We collected drug response microarray data and annotated related drug and targets information from public databases and scientific literature. By selecting top 500 up-regulated and down-regulated genes as drug signatures, we manually established the DrugSig database. Currently DrugSig contains more than 1300 drugs, 7000 microarray and 800 targets. Moreover, we developed the signature based and target based functions to aid drug repositioning. The constructed database can serve as a resource to quicken computational drug repositioning. Database URL: http://biotechlab.fudan.edu.cn/database/drugsig/.

## Introduction

Over the past decades, to develop a de novo drug often takes billions of dollars and about 9–12 years [1]. New drug discovery has grown to be time-consuming and costly. This directly resulted in small quantity and high price of new drugs on the market. Drug repositioning, by exploring new clinical indications for those existing drugs has become an increasingly important strategy for drug development resulted from their proved drug safety and the abridged process of drug discovery and preparation [1–9]. However, traditional drug repurposing is mostly through serendipity or explored from a better understanding of the drugs’ mechanism of action. The efficacy of these methods is very low. When the drug-related and genome-wide data initiatives grew quickly, the mode for computational drug repositioning has been changed.

By integrating data from various sources, like pharmacological, genetic, chemical or clinical data, a set of new computational repositioning strategies and techniques has emerged [3,10,11]. Especially, the Connectivity Map (CMap) [12,13] project which produced large-scale drug response gene expression profiles lead to the establishment and development of methods of ‘gulit-by-association’ and ‘signature reversion‘ for computational drug repurposing [14]. With these methods, Sirota et al. and Dudley et al. had found that an antiulcer drug and an antiepileptic drug can be reused for lung cancer and inflammatory bowel disease by comparing each of these disease signatures to each of the gene expression signatures for 164 drugs from CMap [15,16]. Obviously, the quantity and quality of drug response gene signatures is the core for these computational approaches. But these data still scattered in separated or individual experimental data, it brought about low efficient outputs. So, a database archiving enough drug response gene signatures will be very helpful to computational drug repurposing.

Based on above observation, we collected most of drug response microarray data from GEO or scattered in the separated database to develop the DrugSig database. Moreover, we manually inspected targets information for each drug extracted from microarray data and archived them into DrugSig. Finally, we implemented two functions for repositioning old drugs using signature or target based drug repositioning method respectively. The constructed database will serve as a resource to quicken computational drug repositioning.

## Results and discussion

### Database description

**Fig 1:**
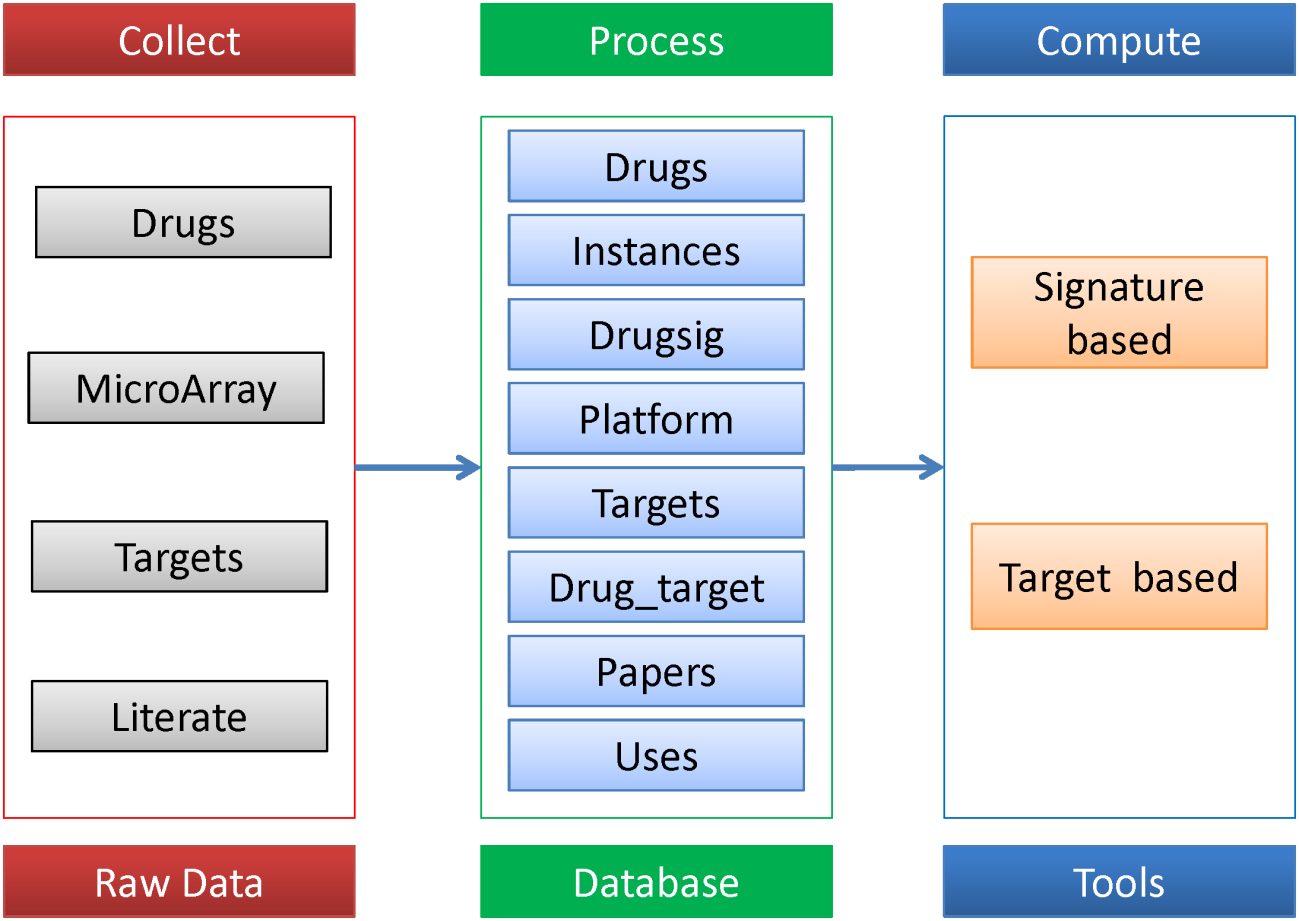
DrugSig architecture. After collecting raw data, we processed them into seven tables in MySql and developed functions to compute drug repositioning by up and down list.

DrugSig was created as a resource for computational drug repositioning utilizing gene expression signatures. As a web based database, DrugSig provides a user-friendly web interface for users to easily query and retrieve information on drug signatures. All the data in DrugSig can be accessed and retrieved directly from the web browser. Fig 1 describes the schema of the creation of DrugSig. All raw data were manually collected from literate and public databases. We processed these microarrays, drugs, targets and literate data into seven Mysql tables, such as drugs, instances, drugsig, platform, targets, drug_target and papers table. On the basis of these data, we developed tools for signature based and target based computational drug repurposing functions and recorded the computational history into uses table.

### DrugSig web interface

**Fig 2:**
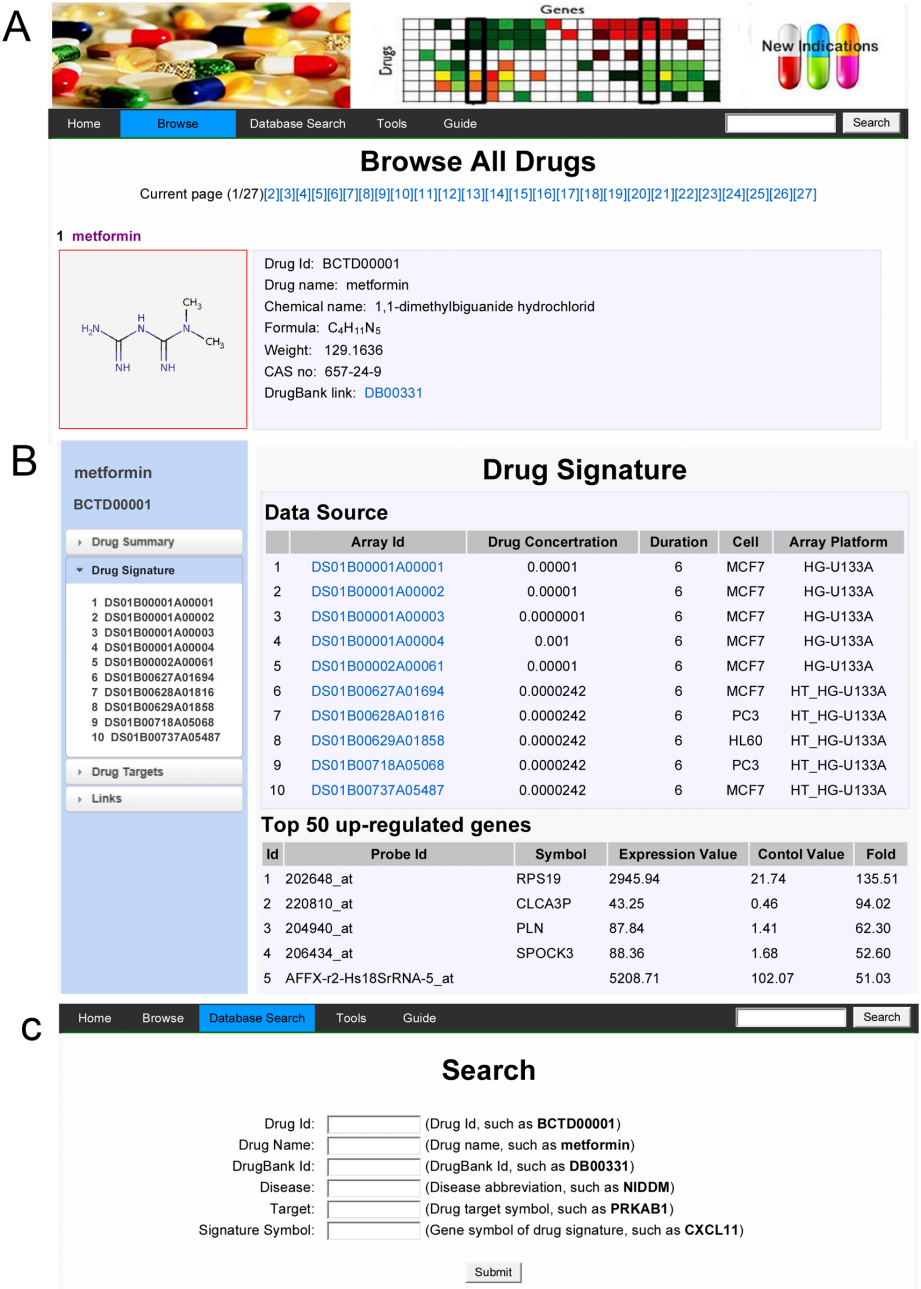
Overview of DrugSig. (A) Browsing the entire database by drugs. (B) A snapshot of the page of drug detail. (C) Searching the database by six options.

A concise navigational interface comprised of the Home, Browse, Search, Tools and Guide options was designed to generate a clearly structured database layout that enables fast and easy navigation (Fig 2A). The Browse interface allows users to navigate all drugs included in DrugSig. The current database is composed of more than 1300 drugs. A click of each drug will display a results page with four sections: drug summary, drug signature, drug targets and links. (Fig 2B). Drug summary section consists of drug name, chemical name, formula, CAS no, description and drug indications. In addition, it also provides a link to DrugBank [17] for further investigation. Drug signature section demonstrates its common signatures which are comprised of top 50 up-regulated and down-regulated genes and its data source (list all related microarray). For each microarray, there is a page to display its signatures. The drug targets section consists of the drug targets and their expression value in cells treated by the drug and other drugs. The prior expression level reflects the expression of the target gene response to the drug while the latter expression level shows the potential of other drugs which inhibit or stimulate the target. The Search interface can be used to retrieve specific information using either a quick or advanced option (Fig 2C). A quick search only allows keywords field, while the advanced search accepts the specification of up to six separate fields: Drug Id, Drug Name, DrugBank Id, Disease, Target, and Signature Symbol. The user can query the database by either one particular condition or a combination of various conditions. The prior five fields search produces a results page which list all drugs meet the specified conditions. The final field search produces a list of the gene expression of specified signature symbol if its expression level lies in top 500 up-regulated or down-regulated genes. The Tools interface implements the signature based and target based drug repositioning functions (see ‘Drug repurposing tools implemented in DrugSig’ section below for further details). The Guide interface provides detail instructions to potential users on how to use DrugSig.

### Drug repurposing tools implemented in DrugSig

**Fig 3:**
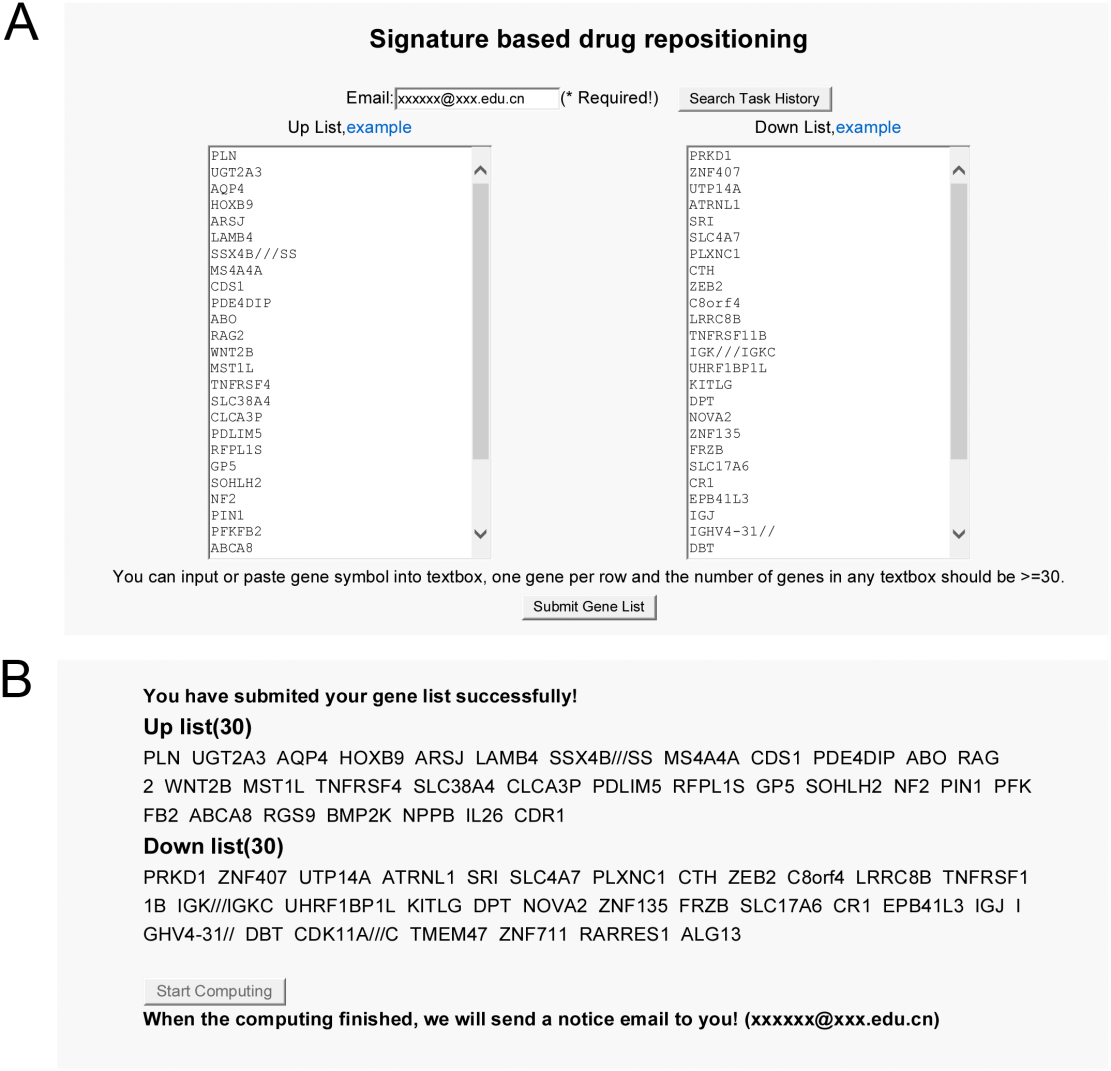
A case study for how to use signature based drug repositioning function. (A) The input interface. (B) The computing interface.

**Fig 4:**
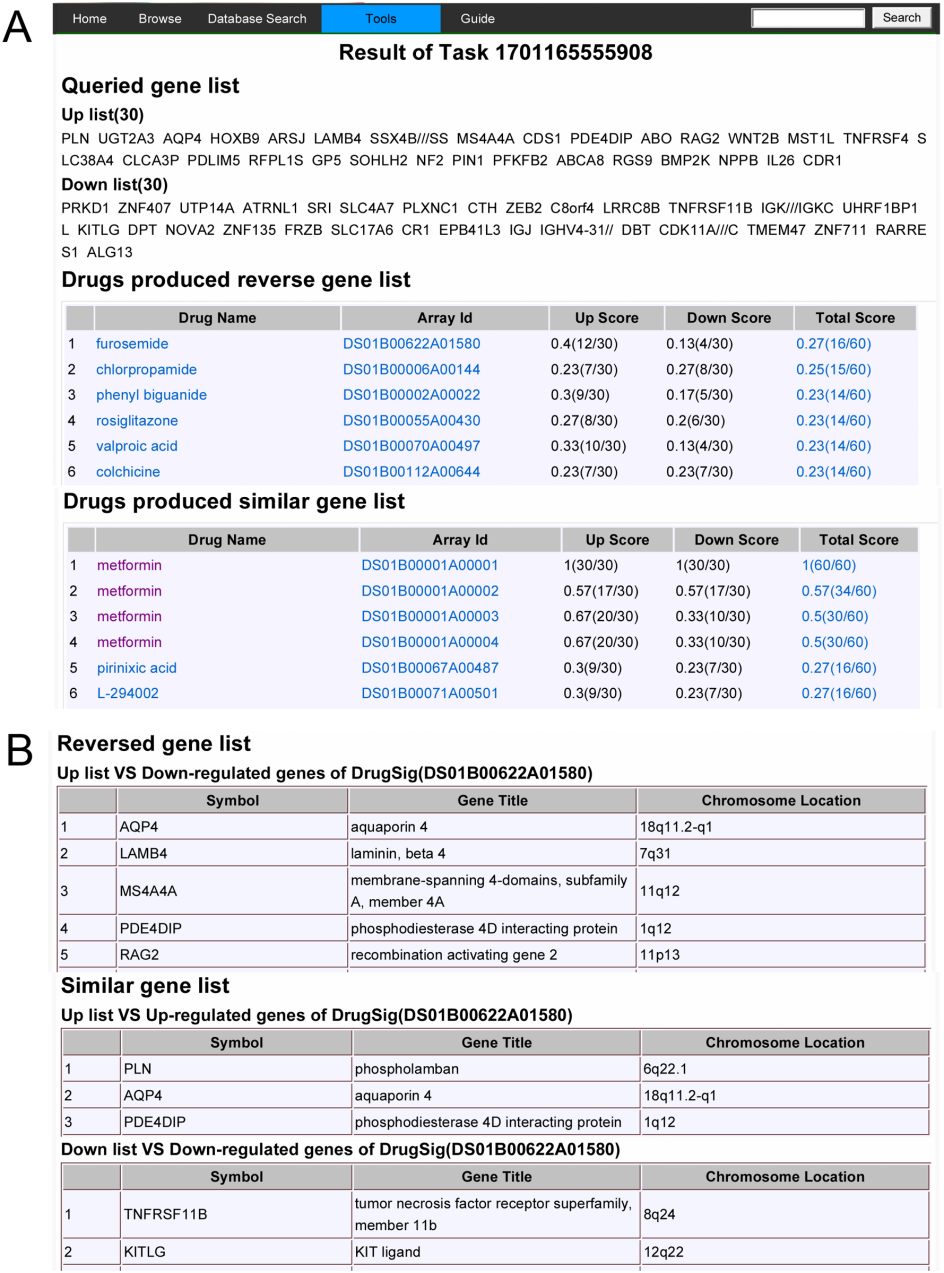
Results of signature based drug repositioning. (A) The drug list for signature based drug repositioning. (B) The gene list for each calculated drug.

Tools of drug repurposing implemented in DrugSig consist of signature based and target based drug repositioning functions. The signature based drug repositioning function provides an interface to input user’s gene list to compute against DrugSig (Fig 3A). After submitting the gene list to DrugSig, user can click the start computing button to compute the scores which is the ratio of the number of common genes between user’s gene list and each gene signature to the number of user’s genes (Fig 3B). Once the computing finished, DrugSig will sent a notice email to user. The results can be accessed later from the email or by searching the task history with user’s email address. The computing result contains queried gene list, top 50 score drugs produced reverse gene list and top 50 score drugs produced similar gene list (Fig 4A). The reverse and similar drugs infer to potential indications. Each drug produces a page display the reverse gene list and similar gene list for further investigation (Fig 4B).

**Fig 5:**
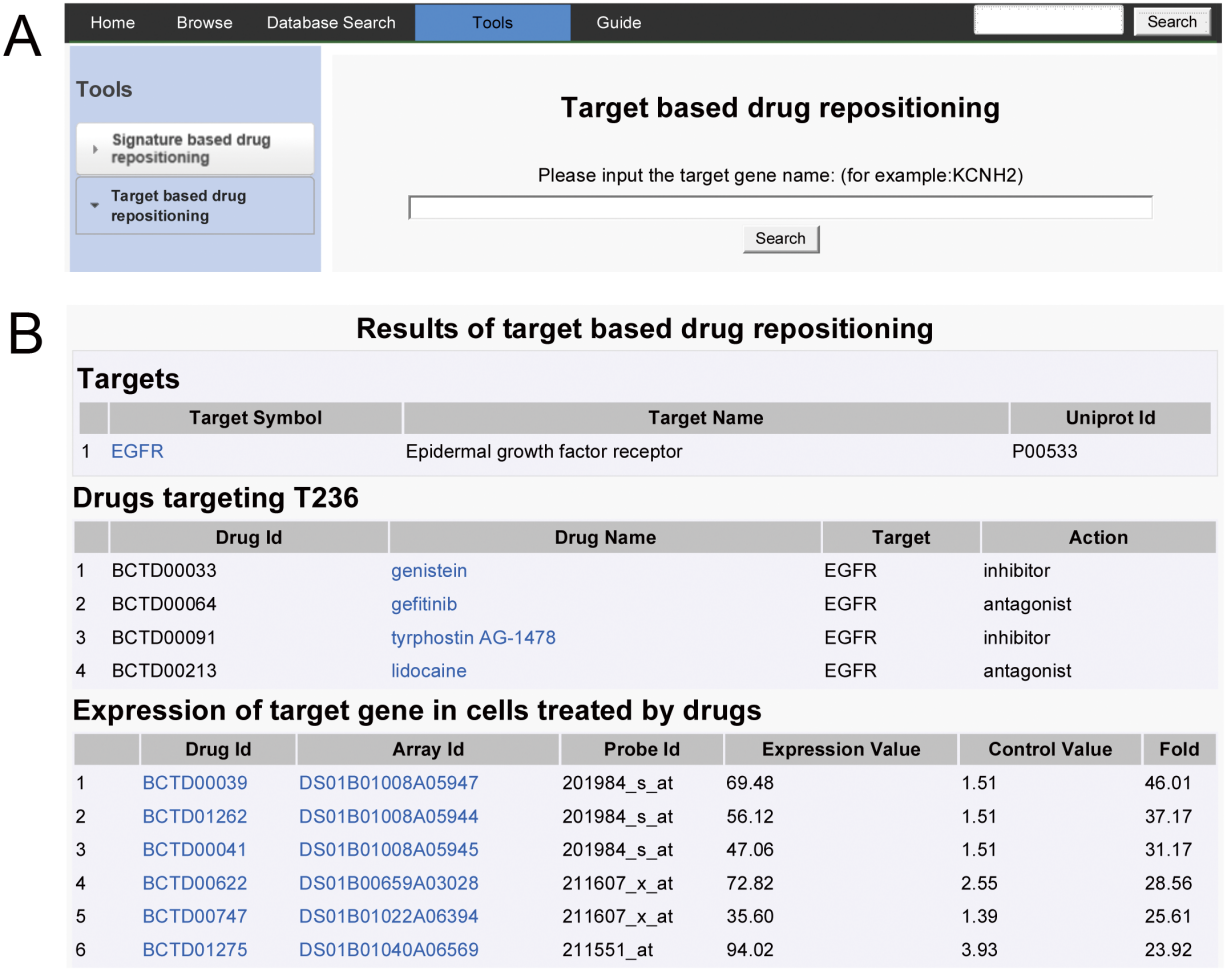
A demonstration for target based drug repositioning function. (A) The input interface. (B) Partial results of target based drug repositioning.

The target based drug repositioning function provides an interface to explore the specified target (Fig 5A) and its targeting drugs, as well as target gene expression level in cells after treated by drugs (Fig 5B). The targeting drugs may have similar indications for drug repurposing. The expression level of target gene partly infers the potential of the drug which either inhibits or enhances the target.

## Discussion

The purpose of establishing the DrugSig database is to aid drug repurposing. DrugSig server as not only a tool to reposition old drugs with user’s input but also an open resource for users to develop new computational approaches for drug repositioning. The current DrugSig contains more than 1300 drugs, 7000 microarray and 800 targets. DrugSig is different from the existing webservers or databases for drug repositioning. Although several drug repositioning related webservers or databases exist such as CMap, PREDICT [18], PROMISCUOUS [19], INDI [20] and Mantra [21], each has certain shortcomings, such as covering a limited collection of drug response microarray or only containing a computational framework. These shortcomings limit the accurate and scope of computational drug repurposing also increase the difficulties in using these data for scientists. Although main data in DrugSig had also been collected from CMap, DrugSig covered pertinent data from other experiments. Moreover, with pertinent experiments growth, DrugSig will contain more and more drugs and signatures.

Moreover, many projects focus on precision medicine will disclose insights between disease and genes. Recently, Rubio-Perez et al. developed an in silico drug prescription strategy based on driver alterations in each tumor and their druggability options and use it to identify druggable targets and promising repurposing opportunities [22]. When applied these insights on DrugSig, we can promptly verify these druggable targets and promising repurposing opportunities. So we constructed a gene list from TCGA Esophageal carcinoma (ESCA) data and submitted it to compute against DrugSig. Results showed that the top compounds predicted to be therapeutic for ESCA were acetysalicylic acid, an anti-inflammatory drug had been reported to treat ESCA, and dizociline, an antiepileptic drug not previously described to have efficacy for ESCA. Related validation work is in progress.

## Limitations and future prospects

Currently DrugSig holds only 1300 drugs and 6000 plus signatures. Moreover, the functions and methods implemented in database are limited. In the future, we plan to updates the data continuously per half a year, and integrate some gene function analysis tools and other computational drug repurposing approaches into DrugSig to improve its interactivity with users and to increase functions to aid computational drug repositioning. In addition, we plan to develop open services convenient for researchers to get gene signatures applied to develop new computational approaches for drug repositioning.

## Conclusions

DugSig is a web accessible database for computational drug repurposing studies. The current version of DrugSig includes more than 1300 drugs, 6000 plus signatures and 800 available targets (till Jan, 2017). The database can be queried either by simply using keywords or by combinatorial conditions searches. DugSig will not only aid in expanding our current understanding of drugs and their mechanisms of action but may have implications in the development of new indications for existed drugs. DugSig now is available at http://biotechlab.fudan.edu.cn/database/drugsig/.

## Methods

### Data acquisition and storage

The microarray data in DrugSig were obtained from the GEO [23] databases or individual scientific researches. The steps of the curation of DrugSig contained collecting, processing and computing (Fig 1). We first searched the scientific literature which contains drug response microarray experiments form PubMed using keywords like “human cell AND treatment AND (‘gene signature’ OR ‘expression profile’) AND (genechip OR microarray OR ‘gene expression’) AND English [la]” and collected available drug response microarray data from GEO database or special sources described in scientific literature. Finally, we obtained more than 7000 microarray raw data. We then read the data via RMA method of affy package in BioConductor [24] and constructed the drug induced signatures using two approaches depending on the quantity of raw data. When the replicates < 3 in raw data we computed the drug signatures by simple fold changes (FC > 2.0 or FC < 0.5) and when the replicates > = 3 we computed the drug signatures by Limma package of BioConductor program (FC > 2.0 or FC < 0.5 and P value < 0.01) which implemented the linear models to calculate the differently expression genes. In addition, if the number of calculated differential expressed probes < 500, we selected all differential expressed probes as signatures. After abstracted more than 1300 drugs from the related experiments, we investigated the drug related information from several public databases such as DrugBank [17,25], KEGG [26], CTD [27] and TTD [28]. Finally, 800 plus available targets were constructed according to descriptions from the literature. All of the collected information and computed data had been classified and filled into seven relational tables in MySQL. Moreover, we constructed the up list and down list file from DrugSig table and used them to implement signature based drug repurposing.

### Database architecture and web interface

DrugSig was built on a 64 bit Windows (2008 R2) server running WAMPSERVER (V2.2d), which integrates the Apache HTTP Server (V2.2.21) with PHP (V5.3.10) and the MySQL Server (V5.5.20). All entries are stored in a MySQL database. The web application was coded in PHP, using the jQuery JavaScript Library (V1.6.2), the Highchart jQuery Plugin, and Cascading Style Sheets (CSS) for the web design. Apache, MySQL, PHP, R and jQuery were preferred as they are open-source software and platform independent, making them suitable for academic use. The web server and all parts of the database are hosted at the Information Office of Fudan University, Shanghai, China.
